# The Role of ACE2 Receptors of the Olfactory System in Anosmia in COVID-19: An Overview

**DOI:** 10.1155/2021/5776801

**Published:** 2021-08-31

**Authors:** Mohammad Javad Nasr, Ali Alizadeh Khatir, Arefeh Babazadeh, Soheil Ebrahimpour

**Affiliations:** ^1^Student Research Committee, Babol University of Medical Sciences, Babol, Iran; ^2^Department of Neurology, School of Medicine, Babol University of Medical Sciences, Babol, Iran; ^3^Clinical Research Development Unite of Rouhani Hospital, Babol University of Medical Sciences, Babol, Iran; ^4^Infectious Diseases and Tropical Medicine Research Center, Health Research Institute, Babol University of Medical Sciences, Babol, Iran

## Abstract

Severe acute respiratory syndrome coronavirus 2 (SARS-CoV-2) is the virus that causes coronavirus disease 2019 (COVID-19). The latest data show that more than 211.7 million people were infected and more than 4.4 million deaths have been reported. The illness presents a wide range of symptoms, ranging from mild to severe. Mild symptoms include cough, fever, dyspnea, fatigue, myalgia and arthralgia, anosmia, and dysgeusia. Furthermore, this virus can affect the central nervous system (CNS) and present a range of mild to severe nervous symptoms, from headache and dysphoria to loss of consciousness, coma, paralysis, and acute cerebrovascular disease. The virus can enter nonneuronal cells of the olfactory epithelium and cause a complete loss of smell. Anosmia and hyposmia are commonly reported in clinics, and being asymptomatic or showing mild symptoms can be primary symptoms in early infected persons. Dysgeusia/hypogeusia is another symptom presented with anosmia/hyposmia. In this article, we reviewed the articles of anosmia and suggested a possible mechanism for this.

## 1. Introduction

The coronavirus (CoV) is one of the major agents that attack the respiratory system of humans and occurs in two forms, severe acute respiratory syndrome (SARS) coronavirus (SARS-CoV) and Middle East respiratory syndrome coronavirus (MERS-CoV). In December 2019, a novel type of coronavirus was first detected in Wuhan, Hubei Province, China, which caused a range of mild to severe respiratory symptoms. The source of SARS-CoV-2 seems to be seafood and wet animals [1–3]. Based on the updated World Health Organization (WHO) report, nearly 211.7 million confirmed cases of infection and about 4.4 million deaths have been reported [4].

SARS-CoV-2 is a beta-coronavirus that belongs to the family of SARS viruses, whose transition path is through person-to-person transmission. Fever, cough, myalgia, arthralgia, fatigue, and dyspnea are the common symptoms. Gustatory and olfactory disorders are commonly reported, and 33.9% of inpatients who were COVID-19-positive had an olfactory or taste disorder [5–8]. SARS-CoV-2 may damage multiple organs and cause several conditions such as respiratory disorder, heart failure, renal failure, liver damage, and central nervous system (CNS) disorder [9]. In fact, in patients with a severe infection, the neurological manifestation includes acute cerebrovascular disease, muscular disorders, and altered consciousness [10]. Nowadays, computer topography (CT) scan combined with laboratory findings such as increased white blood cell (WBC), lymphopenia, elevated C-reactive protein (CRP), and history of exposure have been used in diagnosis of COVID-19, and the reverse transcription polymerase chain reaction (RT-PCR) test is the gold standard for diagnosis [11].

Pathological changes in the lungs are epithelial (such as diffuse alveolar damage (DAD), denudation, and reactive atypical pneumocytes) and vascular (such as microvascular lesions, thrombus, and intra-alveolar fibrin deposits) [12]. In the cohort study by Mostafa Javanian et al., performed on 100 patients with COVID-19, the result showed that most nonsurvivors had comorbidities such as hypertension, cardiovascular disease, chronic kidney disease, and chronic obstructive pulmonary disease. Lymphopenia, elevated C-reactive protein (CRP), and increased level of white blood cell (WBC) were associated with increased death risk [13].

In Tian et al.'s biopsy report on 2 patients with lung cancer, published in February 2020, both patients' lung had exhibited edema, proteinaceous exudate, focal reactive hyperplasia of pneumocytes with patchy inflammatory cellular infiltration, and multinucleated giant cells. These changes appear to target the early stage of COVID-19 infection because the patients had no definite symptoms of pneumonia during this time [14]. In the other autopsy report by Barton et al. published in March 2020, the result was diffuse alveolar damage and chronic inflammation and edema in the bronchial mucosa which appears to be for the late stage of the COVID-19 infection [15].

Our goal in this review article is to collect data to clarify the link between anosmia and COVID-19 and its likely mechanism. For data, we explore the print and preprint papers through the database including Google Scholar and PubMed and the Centers for Disease Control and Prevention (CDC).

## 2. SARS-CoV-2 and Common Symptoms

Respiratory viruses cause mild symptoms in normal people and affect only the upper respiratory tract (COVID-19 can present mild symptoms, too), but in individuals such as newborns, childbearing women, and older people, they can affect the lower respiratory tract and cause severe problems [16]. However, recent reports indicate that severe signs and symptoms can also occur in young adults. Also, studies show that respiratory viruses can affect the nervous system through the respiratory tract [16–18].

COVID-19 might be divided into 2 types, mild, which are found in younger and female patients (anosmia is more common in this type), and moderate to severe (which is seen in older people) [19]. Investigations show that the duration of the illness before hospitalization is about 11 days [20]. Symptoms of COVID-19 range from mild to severe, and the people with COVID-19 may progress to withdrawal within a couple of weeks [21]. There are different reported complications such as dyspnea, cough, fever, dysgeusia and anosmia, ARDS, acute cardiac injury and shock, arrhythmia, fatigue, myalgia, and anorexia. Based on a study of 1099 patients in Wuhan, fever and cough were both common symptoms during hospitalization. In chest computed tomography (CT), the most important findings were ground-glass opacity (GGO) (in 56.4% of patients), and in 17.9%, there were no findings of abnormality [11, 22].

It is confirmed that SARS-CoV-2 can be found in the cerebrospinal fluid and neural damage may manifest in the form of CNS disorders and symptoms [23]. This virus can cause viral encephalitis with symptoms such as headache, fever, vomiting, convulsions, and altered consciousness. Infectious toxic encephalopathy is another nerve condition that presents symptoms ranging from mild to severe. Mild stage symptoms such as headache, dysphoria, mental disorder, and delirium, and severe stage symptoms such as disorientation, loss of consciousness, coma, and paralysis were observed in patients [23]. Also, acute cerebrovascular involvement has been reported frequently which can lead to acute cerebrovascular events such as stroke [23]. Furthermore, SARS-CoV-2 also causes peripheral nervous system (PNS) symptoms such as hypogeusia/dysgeusia, hyposmia/anosmia, and neuralgia [10]. Rothstein et al.'s report from the United States of America (USA) shows that, among 844 hospitalized COVID-19 patients with the conventional vascular risk factor (irrelevant to COVID-19 infection, such as having a history of hypertension or a history of diabetes mellitus) who gave a request for brain imaging, 2.4% had a stroke and 0.9% had intracranial hemorrhage [24]. Another study in Italy by Lodigiani et al. has a nearly similar result of 2.5% of ischemic stroke among 388 patients [25]. In Spain, the study on 1683 patients with COVID-19 by Hernández-Fernández et al. shows that the incidence of cerebrovascular disease was 1.4%, and of these 23 patients, 17 patients had cerebral ischemia, 5 patients had an intracerebral hemorrhage, and 1 patient had posterior reversible encephalopathy syndrome type leukoencephalopathy [26].

## 3. Hyposmia in COVID-19

Hyposmia is a reduction in the sense of smell that occurs because of viruses with the ability to damage or affect the nervous system [27]. This can be an early and mild symptom for COVID-19 patients and could occur suddenly [28, 29]. The study by Bénézit et al. reported that of the 68 patients who were positive for COVID-19, 75% were hyposmic [30]. Another study by Lechien et al. reported that from a total of 417 patients with COVID-19, 357 patients had olfactory dysfunction which was related to infection and among them, 284 (79.6%) had anosmia and 73 (20.4%) had hyposmia [28]. Although hyposmia/anosmia is not the only symptom for the diagnosis of COVID-19, it could be an alert to nursing, isolation, and testing [31].

## 4. Anosmia in COVID-19

Anosmia, the loss of smell, is frequently reported throughout the pandemic and appears to be associated with COVID-19. Dysgeusia is the distortion of the sense of taste and may present with anosmia at the same time [32]. Anosmia with or without fever (>37.5°C) may be a start or perhaps the only manifestation symptom of COVID-19 [33]. This symptom is reported in women more than men, and it is more common in young people [19]. The study by Timothée Klopfenstein et al. reported that 54 of 114 patients with COVID-19 (47%) were anosmic. It also reports that in 85% of patients with anosmia, dysgeusia was comorbid (46 of 54 patients) and in 57% of patients, rhinorrhea was comorbid (31 of 54 patients), but 70% of patients with anosmia had no nasal obstruction [34]. Anosmia is suspected of being a symptom and has been reported frequently in the patients who were positive for COVID-19. Because of reproductive potency in the stem cell of the olfactory system in the nasal cavity, the loss of taste may return in a few weeks [21, 35]. A short duration follow-up investigation by Clair Hopkins et al. reported that of 382 patients, who completed the survey, 86.4% had complete anosmia and 11.5% had a very severe loss of smell at first. But, after a week of follow-up, 80.1% reported lower severity scores, 17.6% were unchanged, and 1.9% were worse [19]. In another study by Joffily et al. done in Brazil on 725 outpatients, 88.8% of patients had anosmia. In other words, this result showed a close link between partial or complete loss of smell and taste and a positive diagnosis of COVID-19 [36].

## 5. Pathogenesis of Anosmia in COVID-19

**Figure 1:**
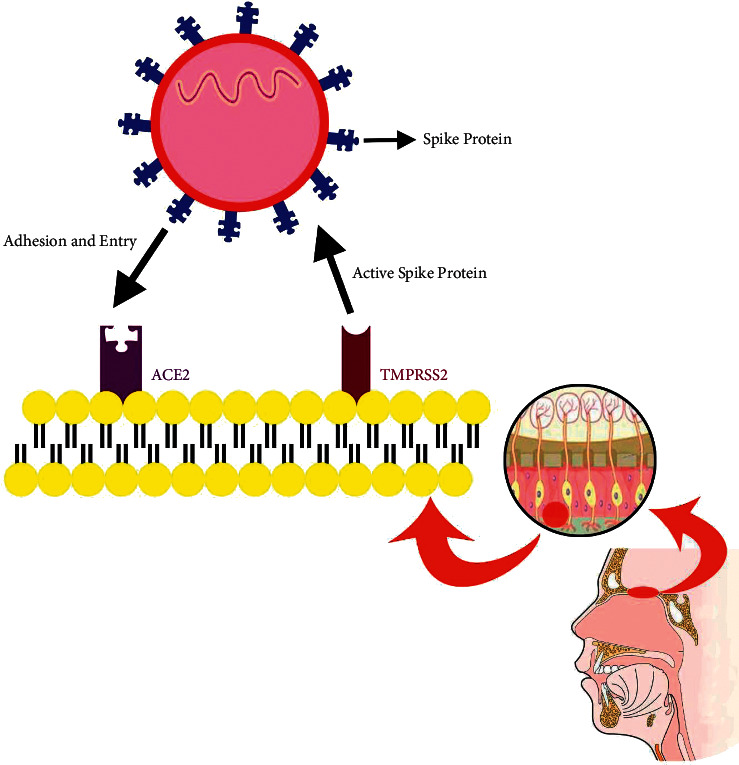
The probable mechanism of SARS-CoV-2 entry into the nasal cavity. TMPRSS2 and ACE2 are receptors existing on the surface of olfactory nonneural epithelium cells. TMPRRS2 activates the spike protein by cleaving viral S-glycoprotein. After that, the virus binds to the ACE2 receptor, and through this way, it can gain entry into human cells.

Loss of smell and taste may be a mild symptom in patients positive for COVID-19, but interestingly, the virological serology test found a high level of viral infection in these patients [17, 19]. COVID-19 can cause demyelination of the brain, and some symptoms such as seizure and distribution of consciousness have been reported [35]. In a study in 2019 conducted by Mao et al., 36.4% of patients had neurological manifestations and these neurological manifestations were more common in patients with severe types of COVID-19 (45.5%) [10]. There is no nasal obstruction or rhinitis in patients with anosmia, and because of this, there may be a link between this virus and olfactory damage in CNS or gustatory receptors in PNS [33, 37]. Aragão et al. reported that of 5 adult COVID-19-positive cases with common symptoms such as fever, headache, and cough, 3 of them had anosmia and injury to the olfactory bulb has been seen in all MRI findings of them [38]. Analyzing datasets shows that angiotensin converting enzyme 2 (ACE2) and transmembrane serine protease 2 (TMPRSS2), which are key genes for COVID-19 virus entry, are not expressed in olfactory sensory cells, instead are expressed in epithelial support cells and stem cells, so smell sense change does not accompany with rhinitis symptoms [39, 40]. In the early stages, SARS-CoV-2 (like other types of CoV) damages the CNS via the olfactory bulb which could likely justify anosmia and dysgeusia [16, 35]. However, the exact mechanism of anosmia is still unknown, but it seems that systemic vascular dissemination and the cribriform plate of the ethmoid bone may be two ways for SARS-CoV to enter the CNS [41]. Firstly, the virus can attack the brain tissue due to neurotropic properties of viruses, but secondly, the virus binds to ACE2 receptors in the capillary endothelium [41]. The expression of the ACE2 receptor in the respiratory airway epithelium is necessary for SARS-CoV to enter and infect the human, and TMPRSS2 activates the spike protein of SARS-CoV and causes infection [42, 43]. As SARS-CoV-2 probably infects through the same way as other coronaviruses, it binds to ACE 2 receptors via the receptor-binding domain (RBD) of the spike proteins. Hence, because the ACE2 receptors are found more in the upper and lower respiratory system and capillary endothelium of the central nervous system, the anosmia can be explained [44–46] (Figure 1).

## 6. Conclusion

There are a wide range of signs and symptoms in COVID-19, from asymptomatic to common symptoms such as cough, fever, myalgia, fatigue, dyspnea, diarrhea, nausea or vomiting, and anosmia in most patients and dangerous situations and death [47, 48]. The reasons that make this virus unique nowadays are increased prevalence and incidence, ineffectiveness and lack of specific drugs to cure, rapid mortality, and unknown future consequences for infected people [49–51]. Anosmia and hyposmia are mild symptoms that are frequently reported [52–54]. Loss of smell may occur due to different reasons such as nasal inflammation, mucosal edema, and airflow obstruction to olfactory receptors in the nasal cavity [55]. Anosmia is thought to occur in COVID-19 via binding of the virus to the host receptors of the ACE2 and TMPRSS2 proteases that express themselves in the nonneural olfactory epithelium [56]. Although anosmia is a mild symptom, since this virus shows its ability to attack CNS, it could be possible to have other serious effects on the nervous system and potentially be dangerous, so probable consequences of CNS infection must be considered. Besides, more research and investigation could explain the exact mechanism of anosmia and be useful in finding the best drugs and treatment to prevent unpleasant events and cure our patients.
